# Microbiomics for enhancing electron transfer in an electrochemical system

**DOI:** 10.3389/fmicb.2022.868220

**Published:** 2022-07-29

**Authors:** Ayush Singha Roy, Aparna Sharma, Bhim Sen Thapa, Soumya Pandit, Dibyajit Lahiri, Moupriya Nag, Tanmay Sarkar, Siddhartha Pati, Rina Rani Ray, Mohammad Ali Shariati, Polrat Wilairatana, Mohammad S. Mubarak

**Affiliations:** ^1^Amity Institute of Biotechnology, Amity University, Mumbai, Maharashtra, India; ^2^Department of Life Sciences, School of Basic Sciences and Research, Sharda University, Greater Noida, India; ^3^Department of Biological Sciences, WEHR Life Sciences, Marquette University, Milwaukee, WI, United States; ^4^Department of Biotechnology, University of Engineering and Management, Kolkata, WB, India; ^5^Department of Biotechnology, Maulana Abul Kalam Azad University of Technology, Haringhata, WB, India; ^6^NatNov Bioscience Private Ltd., Balasore, India; ^7^Association for Biodiversity Conservation and Research Balasore (ABC), Balasore, India; ^8^Department of Scientific Research, K.G. Razumovsky Moscow State University of Technologies and Management (The First Cossack University), Moscow, Russia; ^9^Department of Clinical Tropical Medicine, Faculty of Tropical Medicine, Mahidol University, Bangkok, Thailand; ^10^Department of Chemistry, The University of Jordan, Amman, Jordan

**Keywords:** electroactive bacteria, biofilm, quorum sensing, synthetic biology, microbial electrochemistry, genetic engineering

## Abstract

In microbial electrochemical systems, microorganisms catalyze chemical reactions converting chemical energy present in organic and inorganic molecules into electrical energy. The concept of microbial electrochemistry has been gaining tremendous attention for the past two decades, mainly due to its numerous applications. This technology offers a wide range of applications in areas such as the environment, industries, and sensors. The biocatalysts governing the reactions could be cell secretion, cell component, or a whole cell. The electroactive bacteria can interact with insoluble materials such as electrodes for exchanging electrons through colonization and biofilm formation. Though biofilm formation is one of the major modes for extracellular electron transfer with the electrode, there are other few mechanisms through which the process can occur. Apart from biofilm formation electron exchange can take place through flavins, cytochromes, cell surface appendages, and other metabolites. The present article targets the various mechanisms of electron exchange for microbiome-induced electron transfer activity, proteins, and secretory molecules involved in the electron transfer. This review also focuses on various proteomics and genetics strategies implemented and developed to enhance the exo-electron transfer process in electroactive bacteria. Recent progress and reports on synthetic biology and genetic engineering in exploring the direct and indirect electron transfer phenomenon have also been emphasized.

## Introduction

Microbial electrochemical technologies (MET) is an interdisciplinary concept comprising electroactive microorganisms and electrochemistry employed in bio-electrochemical systems (BES) such as microbial desalination cells (MDC), microbial fuel cells (MFCs), microbial electrosynthesis cells (MEC), and microbial electrochemical biosensors (MEB; Logan et al., 2006; Al-Mamun et al., 2018; Ramírez Vargas et al., 2018; Gomez Vidales et al., 2021; Li et al., 2021). MET employs the electrocatalytic activity of microorganisms to mediate redox reactions at the electrode-electrolyte interface (Rathinam et al., 2019). The common type of interaction observed between the electrode and the electroactive bacteria in BES is biofilms. Biofilms are a group of aggregated microorganisms composed of a single or more than one type of microbes (Lahiri et al., 2019; Kirtonia et al., 2021). In this context, the presence of diverse microbial communities and the synergistic interaction among them tend to play a crucial role (Nag et al., 2021a).

Sessile colonies of the microbial species can adhere to both the abiotic and biotic surfaces *via* extracellular polymeric substances (EPS) and glycocalyx to develop biofilms (Limoli et al., 2015; Lahiri et al., 2021). In this respect, multiple regulatory networks including genetic control are involved in the shift from planktonic to the sessile counterpart. These networks translate information resulting in the change in gene expression, thereby regulating the spatial and temporal rearrangement of the bacterial cells (O’Toole et al., 2000). Moreover, electroactive biofilms (EAB) from such microbes play a pivotal role in electron transfer in BES. Similarly, the formation of biofilms on the electrode surface has a significant effect on the end products (Arunasri and Mohan, 2019). The heterogeneous and dynamic character of biofilms reduces the mass transfer of electron donors and acceptors resulting in reduced catalysis (Lasia, 2018; Wang et al., 2021). Additionally, the thick layer of biofilm limits the diffusion of electron donors/acceptors to electroactive bacteria that cling to the electrode. Thus, biofilm engineering is an important technique for improving microbial electrocatalysis (Rathinam et al., 2019). Several studies have been carried out to find novel electroactive microbes with high electron transfer characteristics, to clearly understand the electron transport mechanisms, and to improve their electron transfer features with special reference to biofilm engineering (Logan et al., 2019; Rathinam et al., 2019). In view of the wide interest in the activity profile of microbiome**-**induced electron transfer activity and due to the enormous application of this technology, this review focuses on the microbiome-induced electron transfer activity and its possible applications, with emphasis on the mechanism of this process.

## Composition and formation of biofilm

Extracellular polymeric substances (EPS), one of the major components of a biofilm, determine the composition and density of the bacterial biofilm. The EPS consists of extracellular proteins (70%), lipids (20%), nucleic acids (5%), and other substances (Branda et al., 2005), and serves as an important component aiding in producing varieties of polymers by the bacterial sessile colonies. Within this context, bacteria such as *Klebsiella aerogenes* produce a limited quantity of polymers (Nag et al., 2021c), whereas the *Streptococcus* species can produce polysaccharides with a wide variety of components (Davey and O’Toole, 2000). Although the polymer helps in cellular adhesion with biotic and abiotic surfaces, it also acts in protecting the cells from a variety of environmental stresses including pH, desiccation, UV radiation, and osmotic shock (Nag et al., 2021b). Other biochemical factors such as oxidation/reduction rates, substrate composition, utilization and substrate specificity, concentration and type of the product, substrate concentration, inoculum concentration, substrate/product inhibition, growth kinetics, and quality affect the biofilm’s adhesion to the substrate (Borole et al., 2011).

On the other hand, change in environment results in the transition of the bacterial species from their planktonic to sessile counterparts (An et al., 1996). The effect of the external stimulus differs from one organism to another and results in the development of the biofilm on various types of surfaces (O’Toole and Kolter, 1998). Some strains of *Vibrio cholerae* and *E. coli* K-12, develop biofilm by the use of a minimal medium with the supplementation of amino acids (Pratt and Kolter, 1998; Watnick et al., 1999), whereas *E. coli* O517:H7 form biofilms only on low-nutrient medium (Dewanti and Wong, 1995). The expression of genes involved in attachment and autoaggregation, as well as others encoding structural proteins, was considerably enhanced in *E. coli* biofilms, according to DNA microarrays. Several outer-membrane generating proteins, including OmpT, OmpC, and OmpF, may be among them; a lipid-encoding protein lpxC is a biosynthetic enzyme; Slp, a protein that encodes an outer membrane lipoprotein triggered by carbon deficiency. In the meantime, genes such as Slp and ompC are linked to the first steps of *E. coli* bacteria adhering to abiotic surfaces in biofilm formation (Prigent-Combaret et al., 1999; Prigent-Combaret and Lejeune, 1999). Gene expression in biofilm cells in *P. aeruginosa* was comparable to the expression of genes in planktonic cells, which would be anticipated to induce quorum sensing and modulate 353 to 616 genes (Martin et al., 2003; Wagner et al., 2003).

Published research showed that several gram-positive infections such as those caused by *S. aureus* and *Staphylococcus epidermidis* are particularly hard to treat with existing antibiotics owing to their high levels of natural antimicrobial resistance. Furthermore, when these organisms develop in a biofilm, they become resistant to high doses of antibiotics that may be delivered (Mathur et al., 2006). Biofilm-related accounts for 60% of nosocomial infections, the majority of which are produced by coagulase-negative staphylococci (Sun et al., 2016; Mohammadnia, 2018). Approximately, the expression of more than 55% of genes at only one of the three-time points indicates temporal regulation of gene expression during biofilm formation. Additionally, glycolysis and the tricarboxylic acid cycle, motility and chemotaxis, phage-related activities, and membrane biosynthesis were among the differentially expressed genes (Sauer, 2003).

### Quorum sensing

The development of biofilm is controlled by a density-dependent communication mechanism known as quorum sensing (QS; Nag et al., 2021a). QS is a bacterial communication method in which cells release, detect, and respond to tiny diffusible signal molecules that regulate the physiological processes of microbial populations (Lahiri et al., 2021). Gram-positive and Gram-negative bacteria have different QS signaling molecules (Table 1). In general, there are at least three types of QS systems: (1) Acyl-homoserine lactones (AHLs) as gram-negative bacteria signal molecules, (2) Oligopeptide-type QS in gram-positive bacteria, and (3) luxS-encoded autoinducer in both gram-negative and gram-positive bacteria QS 2 (AI-2) is used (Li and Tian, 2012).

**Table 1:** Several types of quorum-sensing agents, as well as the species that employ them.

Bacterial species	Chemical substance	References
*Streptomyces* spp.	γ-Butyrolactones	Hassan et al., 2016
Gram-positive bacteria	Oligopeptides	Mull et al., 2018
Xanthomonas, *Xylella fastidiosa*	DSF (cis-11-methyl-2-dodecanoic acid)	Zhu et al., 2002
*Pseudomonas syringae, P. fluorescens*	AHL (N-acyl homoserine lactones)	Noirot-Gros et al., 2018

The QS machinery is important for biofilm formation, exopolysaccharide production, chemotaxis, and motility, all of which are essential for bacteria during pollutant breakdown or detoxification (Mangwani et al., 2016a,b). As a result, genetic manipulation of QS machinery might aid in the development of engineered biofilms with improved degradation kinetics and recent production for application in BESs. The discovery of a link between QS and biofilm design in *P. aeruginosa* sparked a wave of excitement since it offered clear evidence for the function of extracellular signaling in biofilm formation (Davies et al., 1998). However, the QS-regulated genes implicated in matrix formation in *P. aeruginosa* have thus far remained unclear.

On the other hand, QS-controlled rhamnolipid production plays a role in creating the biofilm architecture by keeping the biofilm water channels open throughout matrix development (Davey et al., 2003). The pel and/or psl genes were not revealed as direct targets when a wide strategy was employed to discover QS-regulated genes (Whiteley et al., 1999). However, it is still unclear if the synthesis of any of these polymers is affected by population density in any manner. Thus, the molecular processes underlying the link between QS and biofilm building in *P. aeruginosa* remain unknown. Listed in Table 1 are the QS agents along with the species that employ them.

The relationship between QS and biofilm architecture in *V. cholerae* is well known. VPS stands for the main extracellular polysaccharide expressed in this bacterium’s biofilms (Yildiz and Schoolnik, 1999). The hapR2 is a negative regulator of VPS production; Rugose colonies and thick flow cell biofilms with limited water channels are produced by hapR mutants (Zhu et al., 2002; Zhu and Mekalanos, 2003; Lin et al., 2005). Importantly, LuxO, a two-component response regulator that is most active under conditions of low cell density, indirectly suppresses hapR expression (Zhu et al., 2002). In this regard, at least two QS signals govern LuxO activity, although only the acyl homoserine lactone CAI-1 (cholera autoinducer 1) appears to play a substantial role in biofilm development (Zhu and Mekalanos, 2003). In summary, these findings point to the following surprising model: CAI-1 levels are low enough under low cell density to allow LuxO-mediated regulation of hapR, resulting in VPS synthesis. Thus, this specific bacterium appears to start producing an extracellular matrix while the population density is low, probably before becoming a heterogeneous community.

#### The role of QS in biofilm formation

During QS, cells release autoinducers, which support bacterial colonies in communicating with one another through cell-to-cell interaction. Better production of quinolone results in a 95% increase in the formation of the EPS matrix. Different systems are used in the sensing of a quorum including auto-inducer 2, peptide auto-inducers, and acylhomoserine lactones. There are different ways in which QS affects biofilm formation. Additionally, inducing the concentrations of the QS signals might lead to starvation and stress in the planktonic bacteria population, and bacteria using biofilms, protect themselves from these types of stress. The biofilm environment is stress-free (Monzon et al., 2016).

#### Regulation of quorum sensing in for anaerobic bacteria

The formation of biofilm under anaerobic conditions is highly influenced by QS, and microorganisms require inducers and electron donors for their survival. Under stressful conditions, they adhere to the electrodes, and the thickness of biofilms is controlled by environmental factors having an important role in the formation of biofilm-like electrolytes, applied potential, and QS among other things. QS plays an important role in some facultative anaerobic bacteria like *P. aeruginosa* and *S. enteritidisn*. In this respect, QS affects the anaerobic growth of *P. aeruginosa* PAO1, where the outer membrane proteins, OprF and the rhl QS circuits, are found to be essential for optimal anaerobic biofilm viability (Das et al., 2019).

Research findings indicated that the growth of bacteria is significantly reduced without the functioning of OprF, while lack of rhlR or rhlurel forces the bacteria to undergo suicide from their metabolism due to overproduction of nitric oxide. The effect of QS depends mostly on the rho QS system in the regulation of denitrification. In addition, QS regulates the phenotypes in bacteria, which includes the formation of virulence factors. In this context, the gene expression in *P. aeruginosa* is controlled in response to the cell density-dependent AHL signals, whereas *Salmonella enteritidis* has QS mediated by three autoinducers, AI-1, AI-2, and AI-3. Hence, the QS process is very important for the survival of these anaerobic bacteria, and QS has a very important role to play in anaerobic bacteria.

#### Role of EPS in electron transfer

Extracellular polysaccharides (EPS) have numerous functions in electron transport. Polysaccharides have been traditionally linked with cell-to-cell communication and base connections. Changes in EPS constituents can have functional consequences under which the surface energy is amended and exterior adhesion is altered, thus providing a base for retaining ancillary peptides involved in cell–cell communication, as observed in *Shewanella* sp. (Angelaalincy et al., 2018). In this perspective, investigations with *Geobacter sulfurreducens* have been the focus of attention, demonstrating the importance of EPS as adhesion sites for ancillary oxidation/reduction polypeptides, enabling multi-cellular populations to transport charged particles. Mutants lacking the gene encrypting exopolysaccharide matrix yield ceased to form ion transport biofilms on anode and cathode. Along this line, *G. sulfurreducens* can generate external tethering polymers with c-type cytochrome receptors, which are required for energy transport to the electrode (Rollefson et al., 2011). As a result, EPS is an important element not just in bacterial growth but also as an electron transport medium in MFC.

The ability of electrocatalytic bacteria to engage in external energy transfer is the barrier to BESs. Microbes act as energy transmission vehicles in this exoelectrogens transfer (EET) operation, through direct or assisted modes. Direct EET occurs when an electrode, mostly the anode, and the electroactive bacteria directly interact using transmembrane proteins or electroactive microbial sites. Endogenously (e.g., phenazines) or extracellularly accessible (e.g., methylene blue, neutral red, AQDS, flavins, etc.) intermediary compounds, also known as electrochemical carriers, and ferry energy across cells to the anode through the external watery media, function as intermediaries in assisted EET. Influenced cross-species transfer of electrons, or MIET, arises when one population produces compounds or mediators that are eaten by some other creatures in a community.

A thorough examination of microorganisms found in electrogenic multilayer colonies sheds light on the mechanisms that transform complicated organic materials in effluents to electrical energy in BESs (Kiely et al., 2011). Direct interspecies electron transfer (DIET), which occurs among microbes or in affiliation with electromechanical conductive components, has also been discovered in the last century. It can be induced in designed organizations for enhancing sewage treatment objectives and for regenerative braking in bio-electrochemical innovations (Cheng and Call, 2016). Hence, the contribution of extracellular polymeric substances of biofilm matrix formed by microbial cells in BESs becomes the focal theme for researchers.

#### c-di-GMP signaling molecules

**Figure 1:**
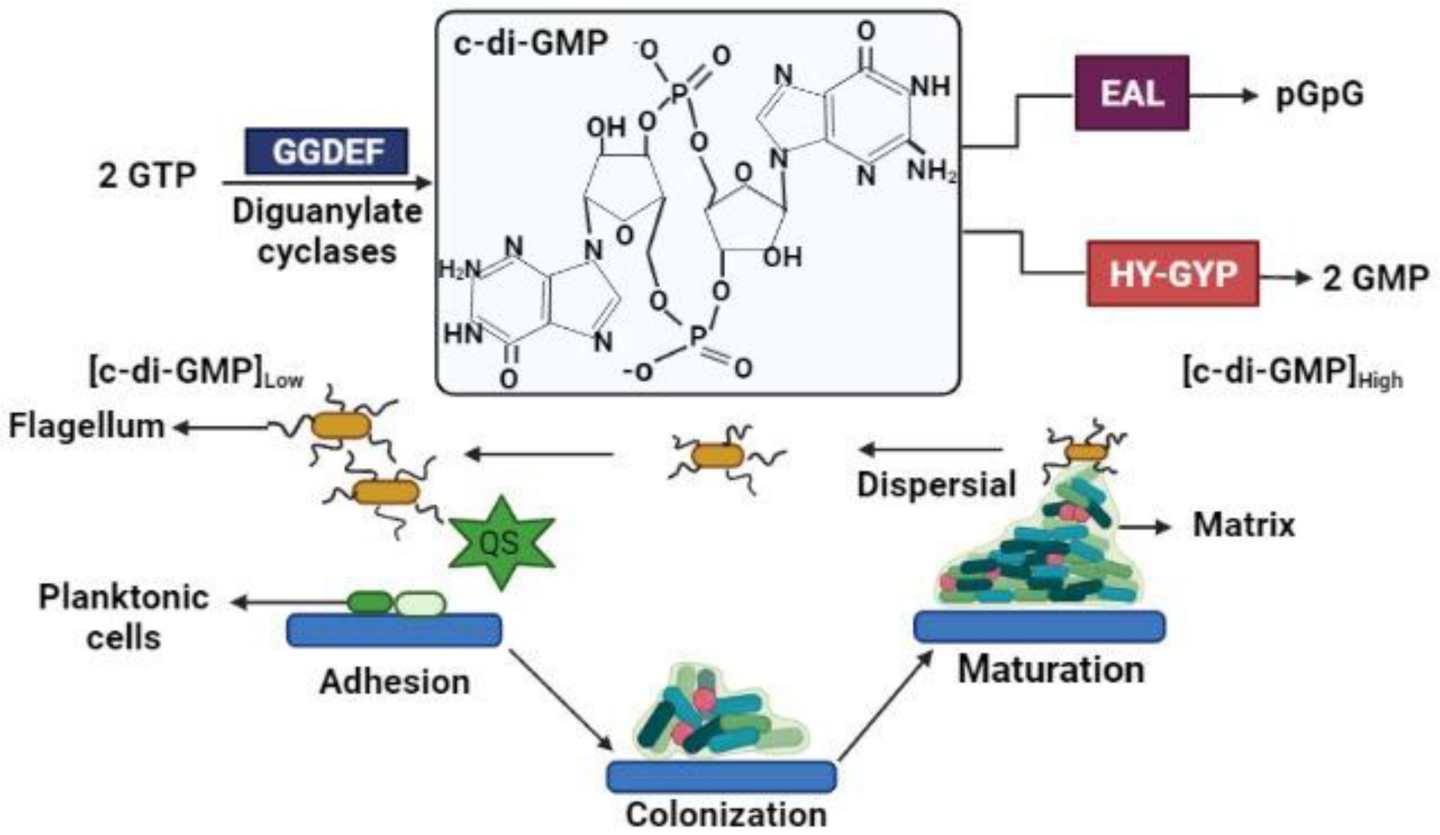
c-di-GMP signaling in biofilm.

Some secondary messengers in bacteria help in the coordination of various cellular processes including the growth of bacteria and other properties such as virulence, formation of biofilm, motility, and the progression of the cell cycle. One of the main secondary messengers is the bis-(39e59)-c-di-GMP (Figure 1), which is a signaling molecule that is synthesized by a different bacterium during its transformation from platonic lifestyle to a sessile bacterial biofilm formation and *vice-versa* through the dispersal of biofilm. The c-di-GMP manipulates the formation of biofilm and the dispersal, which are done through various types of genetic interactions. Findings showed that bacterial cells like *P. aeruginosa* and *E. coli* demonstrate the correlation between concentrations of c-di-GMP compared to the biofilm formation or the dispersal (Andersen et al., 2021).

## Development of biofilm on the electrode surface

In recent decades, a novel type of bacterial structure known as electroactive biofilm (EAB) has been found to grow randomly on charged electrodes. On a typical electrode surface, EAB conducts electrons over extended distances and achieves quasi-reversible electron transfer (Prévoteau and Rabaey, 2017). EABs have comparable temporal development, composition, and structure to traditional/natural biofilms, but they have different electrochemical characteristics since they are produced on electrode surfaces that function as electron sinks to receive electrons. Microbial attachment to a biotic or abiotic surface is the crucial starting point for biofilm development and is affected by a variety of physical variables. In this regard, physiological factors which determine adhesion include surface mechanical characteristics, biofilm energies along with solid substrate surface energies, chemical reactions, and electrostatic interactions (Arunasri and Mohan, 2019). The capacity of bacteria to give electrons, either directly as observed in *Geobacter* sp. and *Shewanella* sp. or indirectly through mediator-assisted charge transmission as in *Pseudomonas* sp., etc., facilitates the initial adhesion of the electroactive bacteria onto the electrode surface. BESs, such as MDCs, photo microbial fuel cells (photoMFCs), and MECs, have proven the capacity of electroactive bacteria to form biofilms on the electrode surface (Arunasri and Mohan, 2019; Rathinam et al., 2019). Similarly, extracellular polymeric matrices that assist in surface modification and mediate adhesion between surfaces and microorganisms may alter surfaces into the biocompatible form using their inherent processes. However, a charge transfer barrier along the electrode-electrolyte interface in BES does not increase by such changes (Flemming and Wingender, 2010).

**Figure 2:**
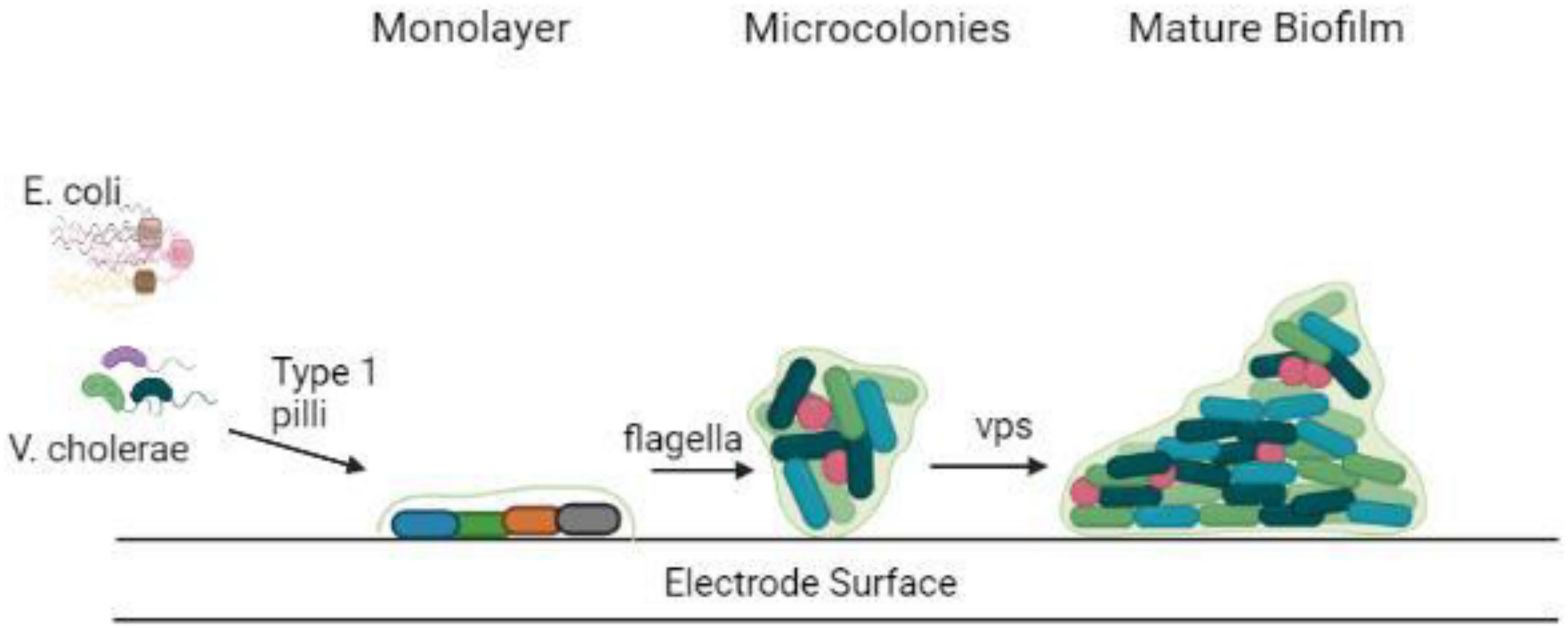
Attachment of biofilm on the electrode surface.

Electrogenic bacteria form biofilms that differ from non-electrogenic bacteria in several ways. The existence of EET elements including their pili, c-type cytochromes, or endogenous electron mediators separates conducting and non-conducting biofilms. The presence of an external electron acceptor, the electrode on which biofilm forms and the electrogenic bacteria that attach to the electrode surface and respire by giving electrons are among the other variables (Semenec and Franks, 2015). On the other hand, the electrodes employed in these BESs not only function as an electron sink but also promote adhesion, which helps to enhance the electrochemically active bacteria. Therefore, electrogenic bacteria predominate in the conducting biofilm produced on the working electrode, as opposed to planktonic cells (Dheilly et al., 2008; Sun et al., 2016). Electrogenic bacteria choose electrodes over many other electron acceptors in BESs because the anode potentially boosts the Gibbs free energy obtained by the microorganisms over other electron acceptors (Lian et al., 2016). *G. sulfurreducens*, *Geobacter metallireducens*, and *Shewanella oneidensis* were among the pure cultures studied for their capacity to produce conductive biofilms on anode surfaces (Ringeisen et al., 2006), *T. potens*, *Thermincola ferriacetica* (Rubaba et al., 2013), *P. aeruginosa* (Guo et al., 2015), *E. coli*, and *D. desulfuricans* (Semenec and Franks, 2015). Furthermore, mixed bacterial biofilms, produce higher power density than pure culture biofilms (Guo et al., 2020). Depicted in Figure 2 is the attachment of biofilms on electrode surfaces.

The metabolism within the biofilm is dependent on the microenvironment created by the co-existence of various bacterial species residing within it (Figure 2). For example, the interplay of fermentative and acidogenic bacteria degrades complex organic materials into molecular hydrogen (Davey and O’Toole, 2000; Annie Modestra and Venkata Mohan, 2019). The microenvironmental niche inside biofilms provides an excellent condition for the formation of symbiotic interactions between bacteria. Moreover, the development of colonies inside the biofilm is aided by the interactions between metabolically cooperative bacterial populations. Based on dietary content and metabolites swaps, colonization could be formed from one or multiple microbial species (Donlan, 2002).

### Biofilm formation on electrode materials

In any electrochemical cell, the electrode material is the most important factor. Electrocatalysts are usually immobilized on electrodes; however, in other situations, the electrode material functions as a catalyst. The electrode material must have a large surface area, high conductivity, low resistance, and low impedance. Additionally, the electrode material must be compatible and help in the adhesion, development, and multiplication of microorganisms on the electrode (Rathinam et al., 2019). Non-biocompatible and poisonous electrode materials can accept and transmit electrons, and promote electron transfer processes *via* electron shuttling chemicals, but they may not efficiently mediate the transfer of electrons and biofilm development. However, they can be engineered to facilitate direct electrochemical reaction processes with the right surface engineering methods (Nishio et al., 2010; Li et al., 2017; Ter Heijne et al., 2021).

#### Carbon/graphite electrode

For bio-electrochemical applications, carbon materials such as graphite, carbon felt, charcoal foam, carbon cloth, carbon paper, and carbon brush have been broadly employed (Li et al., 2017). Various physiological features and physical properties have already been observed in such carbon compounds. These features enable microorganisms to mediate high-speed electron transfer processes and, therefore, are regarded as an interesting category of electrode materials for microbial fuel technologies. In this respect, biofilm and interfacial colonization substantially increase when carbon compounds are used, leading to improved microbial catalysts. Numerous other materials such as nickel foam, stainless steel, and titanium among others have also been used and documented. According to recently published research, bio-anode performance may be graded in the range of uncoated titanium > Pt-coated titanium > flat graphite (Ter Heijne et al., 2021).

#### Titanium electrode

Although titanium has high resistance to corrosion, poor conductivity, scalability, and low bioactivity make it unsuitable for bio-electrochemical uses. Anodic biofilm development is much easier when surfaces are functionalized by putting TiO2 nanotubes on them. In photo- bio-electrochemical devices, TiO2 is commonly employed because of its wide bandgap. For example, graphite felt, carbon cloth, and paint do not appear to be viable substances for the formation of photosynthetic biofilms, whereas the creation of photosynthetic biofilms was successfully demonstrated using carbon barrier and carbon sheets (He et al., 2009; Horie et al., 2009).

#### Ceramic electrode

Research findings indicated that porous ceramic electrode materials have been used for MFC biofilm development (Thorne et al., 2011). Among these were fluorine-doped tin oxide (FTO)-coated nanoporous TiO2 ceramics, utilized to produce biofilms of *Chlorella vulgaris*. There were no biofilms formed on the graphite fiber electrodes when *C. vulgaris* was used. A fibrous external network was present on the FTO-coated ceramic electrode, which allowed *C. vulgaris* to adhere to the electrode. As a result, the extracellular membrane was less fibrous. Furthermore, studies on biofilms formed on carbon felt wires revealed deformed cells and a lack of extracellular matrix, both of which are essential for biofilm development and persistence. There was a dense cluster of *C. vulgaris* cells inside the carbon felt material, however, they did not attach very well to the wire surface.

#### Metallic electrode

Metal electrodes such as copper, gold, and silver, as well as stainless steel, nickel, cobalt, and titanium, were studied in-depth (Baudler et al., 2015) to build microbial bio-electrochemical reactors (Table 2). A polarization potential of 0.2 V was used to produce biofilms on carbon, gold, and silver anode materials, whereas a potential of −0.2 V was employed to grow biofilms on copper, steel, nickel, and titanium. According to bio-electrocatalytic studies, gold, silver, and copper showed the best performance among the metals examined, with current densities of 1,175, 1,119, and 1,515 mA/cm^2^, respectively. Because of their biocompatibility, gold, silver, and copper have greater current densities than other metals. In comparison to graphite, numerous metals have a transmission that is 1–2 times of magnitude greater. On the other hand, stainless steel’s decent price and excellent conduction make it an appealing resource for microbial fuel purposes, particularly in terms of economic feasibility. Unfortunately, due to their low affinity, microbiological development on stainless steel electrolytes remains severely restricted, and thermal oxygenation was used to strengthen bioactivity.

**Table 2:** Overview of specific current density and thickness on electrodes of different electrode materials (Baudler et al., 2015).

Electrode material	Current density (mA/cm^2^)	Thickness on electrode (mm)
Gold	1175	127 ± 11
Silver	1119	154 ± 10
Copper	1515	249 ± 21
Graphite	984	117 ± 13
Nickel	384	77 ± 9
Stainless steel	674	–

The electrolytic preprocessing using graphene sheets enabling better biofilm construction has indeed been described throughout research. An exfoliation on graphite’s top produces carboxyl-containing side chains that increase colony adhesion only at the active material junction and, therefore, enhance electrochemical properties. The oxide layer of graphene typically includes atmospheric O_2_, resulting in the development of various reactive compounds onto the graphene sheets layer, including phenolic, carboxylic, acetic, as well as quinoline, that improve charge separation across the biofilm junction (Cercado et al., 2013). In this respect, the supplied electrical voltage affects the microbiological biodiversity of the film. Growing advancement also sparked the application of such multiple (3D) sensors, which offer numerous benefits including a wide given surface for bacterial adhesion or nanoporous geometries for basal dispersion. Such characteristics, throughout practice, lead to significantly increased bio-electrocatalysis as well as microbial fuel efficiency and reliability.

### Mechanism of electron transfer from biofilm to the anode

**Figure 3:**
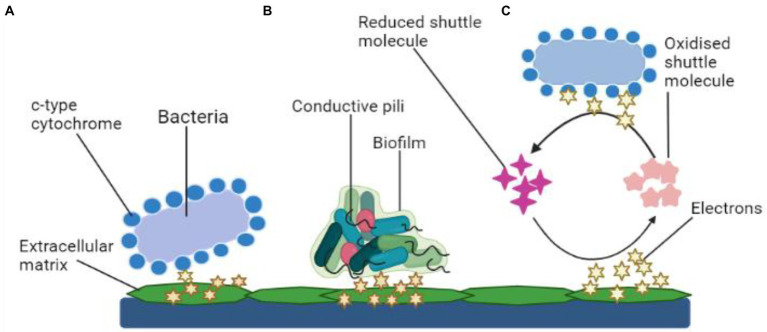
Mechanisms of electron transfer. **(A)** Electron transfer *via* membrane-bound c-type cytochrome, **(B)** Mechanism of electron transport using bacterial pili, and **(C)** Transfer of electrons using shuttle molecules.

There are two kinds of power generating mechanisms in MFCs: direct and indirect electron transfer as depicted in Figure 3.

#### Direct transfer

Microbes simply transmit electrons through their cell wall to the electrodes. The transmission is facilitated by polypeptides adsorbed on the cell membrane (cytochrome). Common types of electroactive bacteria that follow this mechanism are *Rhodoferax ferrireducens* and *G. sulfurreducens* (Angelaalincy et al., 2018). The direct transfer of electrons requires physical interaction between the microbial surface containing EPS or even the membrane organelle and the fuel cell electrodes, which is usually an anode (Angelaalincy et al., 2018). Such electron transport mechanism does not include any diffusive oxidation/reduction species. Only an electrochemically active microbe can transfer electrons from its cell surface to the electrodes (Matsena and Nkhalambayausi Chirwa, 2022). Due to the non-conducting behavior, living organisms are often thought to be electrically inert. Moreover, research findings have shown that a microorganism with a surface electron transfer polypeptide enclosed within an EAB may effectively employ the technique. However, channel proteins control the electron flow from within cells to the surface, while outer-membrane (OM) oxidation–reduction factors control the flow of energy to an exterior, concrete final electron recipient, such as an electrode. Several metal-reducing bacteria present in the soil such as *Geobacter, Rhodoferax*, and *Shewanella*, have c-type cytochromes, which are multi-heme complexes present in such microbes also with EAB (Limo et al., 2018).

As previously mentioned, DET necessitates a physical connection between EAB and their cytochrome. However, at the anode, the topmost covering of microorganisms in the film will be electrically functional (Kiran and Patil, 2019). As a result, the bacterial population in the very first multilayer measures the MFC efficiency. The thickness of the colony increases with the aging of the cultures and the production of more EPS. Consequently, it has been observed that the existence of EAB communities with thick monolayers has a substantial impact on microbial fuel cell efficiency. Aside from cellular proteins, microbes contain adhesion fimbriae—pili, which are made of amino acids and sortase enzymes. In this respect, several *Geobacter* and *Shewanella* cultures have been shown to generate pili with electroconductivity (Mandlik et al., 2008). Such pili, also known as nanowires, connect to the cell’s membrane-bound cellular proteins, allowing for electron transport to the cell’s margins. The nanostructures also prevent the synthesis of denser EA biofilms, which improves anode efficiency.

#### Indirect transfer

For charge transfer, an electron mediator is required in an indirect mode. Within this context, secondary metabolites (intracellular redox intermediates) are especially fascinating in indirect transmission because their production allows electrochemical reactions to occur without the influence of environmental redox messengers. Mediated-electron transfer is the term used to describe this process (MET). Microbial-produced soluble electron transporters such as pyocyanin and flavins are considered potent mediators. The mediator works as a bidirectional electron acceptor, moving atoms from the microbial cell to a substantial oxidizer or into biofilm sheets, where they get re-oxidized and become accessible for future redox reactions (Umar et al., 2021). Thus, the bacterium can deal with particles at suitably increased levels by producing minuscule quantities of such chemicals (specifically in the anodic film). *P. aeruginosa* pigmentation pyocyanin, for instance, was already discovered as essential for the bacteria’s electrochemical conductivity (Muller and Merrett, 2014).

In comparison to MFC without mediators, quinone-mediator (2-amino3-dicarboxylic-1,4 naphthoquinone) generated from *S. oneidensis* can enhance the energy capacity of MFC by a factor of two (Schröder, 2007). The microbe *Pseudomonas alcaliphila* also can generate electrochemical intermediates by itself. Apart from electrochemical intermediaries, by-products obtained by microbial activity can potentially assist the indirect electrochemical reactions through oxidative stress of the generated by-products. In both instances, transmission of the charged particle occurs *via* microbe interaction with the electrochemical cell, whether in a straightforward way or *via* dissolved shuttles including ubiquinones, colorants, tints, and nanoparticles that shape reversible electrochemical couples, which are dispersible and non-toxic to the various microorganisms, physiologically non-degradable, and durable in all the oxidized and reduced types (Aghababaie et al., 2015).

### Electrochemically active biofilm modeling

Core concepts for understanding the external transfer of electrons are anticipated to be provided through numerical techniques. Initially, the EAB modeling study is focused on equations built specially to explain the functioning of MFCs and BESs: the objective was to connect bacterial growth with MFC energy output and maximize these activities based on numerical estimates. The latest ones now concentrate on the intricate extrinsic energy transfer pathways seen in EABs. The algorithms’ major aims are to forecast power and link it to electrons and protons transport.

#### Current generation prediction from electrochemically active biofilms

Estimating power production from developing EABs using both regulated and resistive electron transport processes was a technique to validate practical outcomes found in research (Table 3). In this respect, two equations were generated to accommodate for biofilm development rate and load transfer within microbes throughout power production. Both models demonstrated that all facilitated charge transfer (modeled by Butler–Volmer kinetics) and direct charge flow (modeled by the Nernst-Monod equation) were conceivable. Moreover, research findings on the limits of power production due to energy transfer offered a logical scientific foundation for designing conductors for application in BESs (Rismani-Yazdi et al., 2008). However, additional development of the systems to encompass the effect of pH and more complex biological pathways would be required in the future.

**Table 3:** Various techniques to enhance the functioning of MFC by improvising the mechanism of EET.

**Microbial species**	**Investigated approach**	**Synopsis**	**MFC productivity**	**References**
*Geobacter sulfurreducens*	Excision of GSU1240	PilZ-domain protein and augmented biofilm synthesis	A 50% more yield of current and 70% increase in power density	Leang et al., 2013
*Synechococcus elongatus*	Expression of OmcS from *G. sulfurreducens*	Enhanced direct EET	Caused a 9 times increase in current production	Sekar et al., 2014
*Shewanella oneidensis*	Excessive expression of D-lactate transporter	Increased substrate transfer and 61% more metabolism of D-lactate	Caused a 1.3 times increase in current production	Zhu et al., 2017
	Disorganization by random transposon installation of uvrY	Minimal expression of genes associated with the synthesis of exopolysaccharides and greater adhesion of cells on the anode	Caused 60 to 90% increase in power yield	Kouzuma et al., 2010
	Suppression of UvrY and expression of SpeF *via* CRISPRi	Due to the suppression of both UvrY and SpeF, biofilm development is raised by 2.3 times	Caused a 1.7 times greater current yield	Cao et al., 2017
*Escherichia coli*	Expression of CymA and MtrCAB from *S. oneidensis*	The native *E. coli* cytochrome maturation framework CcmABCDEFGH was expressed	Caused a 4 times increase in power output	Jensen et al., 2017
	Omission of ldhA	LdhA increases the ratio of intracellular NADH/NAD^+^ by two times	Caused a 6 times increase in power output	Yong et al., 2012
*Pseudomonas aeruginosa*	Excessive expression of PqsE in a *P. aeruginosa* pqsC strain	Improved synthesis of phenazines	Caused a 5 times increase in the current density	Wang et al., 2013
	Excessive expression of the rhl QS framework	Higher synthesis of the QS signal molecule BHL and increased synthesis of phenazines	Caused a 1.6 times increase in the current density	Yong et al., 2011
	Excision of retS	Improved synthesis of phenazines	Caused a 45 times increase in current output	Venkataraman et al., 2010
	Expression of IrrE from *Deinococcus radiodurans*	Heterologous expression of IrrE improved phenazine synthesis and minimized internal resistance	Caused a 71% increase in the current yield	Luo et al., 2018
*Saccharomyces cerevisiae*	Surface exposal of glucose oxidase (GOx) from *Aspergillus niger*	GOx oxidizes glucose and emits electrons	Caused a 1.9 times increase in MFC functioning	Fishilevich et al., 2009
	Surface exposal of cellobiose dehydrogenase (CDH) from Corynascus thermophilus	CDH oxidizes numerous sugars and transports electrons directly to an anode	Caused a 12 times increase in power output	Gal et al., 2016

The following equation gives the usual version of the Butler–Volmer Equation:


(1) $$I={\mathrm{nFk}}^{o}\;\left(X_{\mathrm{red}}e^{\left(1-x\right)f(E-\mathrm{Eo}}{{}_{x}}^{)}–X_{\mathrm{ox}}e^{-\alpha f(E-Eο}{{}_{x}}^{)}\right)$$

where *n* is the number of electrons transferred, *I* is the current density (A m^−2^), *k^o^* is the typical heterogeneous rate constant (ms^−1^), *X_red_* is the quantity of the reduced state of the redox pair at the biofilm-electrode interface (mM), X_ox_ is the amount of the oxidized state of the redox pair at the biofilm-electrode interface (mM), α is the charge transport constant (unitless), *F* is the clustered element of the Faraday constant, *f* (s A mol^−1^), *T* is the temperature (K), and R is the universal gas constant (J/K mol). *E* represents the biofilm electrode voltage (V) and *E^ο^_x_* is the redox couple’s typical reduction potential (V). Listed in Table 3 are the various techniques used to enhance the functioning of MFC.

The Butler–Volmer formula is suitable for representing assisted electron transport processes since it connects the quantity of the electron facilitator at the biofilm electrolyte interface and the colony electrolyte voltage to power generation. This formula is frequently combined with the transport of electron intermediaries *via* biofilm to give a technique for estimating the power and layer spectra of intermediary amounts. For BESs, several of the factors in this formulation are important. The typically mixed-flow variable, for example, has been shown to govern the temperature reliance of power in sedimentary MFC. This factor is determined by the biofilm electrode microstructures as well as the presence of electrochemical couplings in the unit. Characteristics of the redox couple affect the electron transport coefficient as well (Babauta et al., 2012). Finally, the typical redox potential is crucial for EAB modeling since it may determine the quantity of power accessible to a microbe as well as which end electrophile the bacteria can employ. To simulate conductive charge flow, the Nernst-Monod equation can be employed:


(2) IImax=11+e−fE−EkAkks+s

where I_max_ (A m^−2^) is the maximum electrical potential, E_KA_ is the voltage that is half that of the limiting power (V), S is the amount of the charged particle substance (mM), and Ks is the Monod half-saturation constant (mM). This formula is a type of nonlinear Monod equation in which the electron receiver is a substantial electron receiver that may be reached through immediate transmission rather than a dissolving acceptor substance. E_KA_ is the crucial factor in this calculation since it determines where the curvature peak appears in an optimum slow-scan CV.

#### Predicting exogenous electron transport mechanisms in EABs

In contrast to the comprehensive mathematical analysis of the whole anode, the practical current–voltage dependency in EABs has been studied. The Butler–Volmer–Monod concept (Equation 3) was created to predict bio-anode polarization arcs and physiological dynamics as a consequence of voltage and feed quantity (Zeng et al., 2010). The Butler–Volmer–Monod model is described as follows:


(3) $${}^{\prime }s\frac{I}{Imax}=\frac{1-e^{-f(E-E_{s/p)}}}{K1e^{-f\left(1-\propto \right)(E-E_{s/p)}}+K2e^{-f(E-E_{s/p)}}+\frac{K_{M}}{S}+1}$$

where K1 and K2 are variables that are clubbed together (unitless), E_S/P_ is the isothermal electrode potential, and K_M_ is the substrate attachment coefficient (mM). It is worth noting that the substrate attachment variable in the formula is not the same as the Monod partial concentration variable. Analysis indicates that the actual Monod coefficient is a result of anode voltage, and that Km is just comparable to KS at reasonably great excitation energies (high E-E_S/P_ ratios). Likewise, K1 and K2 seem to be tricky concepts, which appeared to be solely dependent on the electrical voltage and the microbe in question. K1 is the frequency of metabolic source consumption divided by the frequency of electrolytic interchange power density, while K2 is the frequency of output synthesis divided by the frequency of source production within the microbe. Furthermore, the above analysis reveals overall ion transmission from molecules to the biofilm layer seems to be the major barrier to the production process, irrespective of whether the electrochemical reaction pathway is facilitated or resistive. The external charge transfer method developed has shown some intriguing limits in the interpretation of empirical results.

To offer crucial data, EAB systems must convey complete images of exogenous energy transfer, biological mechanics, communal connections, epigenetics, and hydraulics (Mahadevan et al., 2011). Additionally, results must be interpreted from the perspective of existing statistical equations. Models are likely to persist and be developed as a result of the increasing number of innovative sophisticated approaches being used. Unfortunately, the majority of these models lack the necessary empirical values to be evaluated. Furthermore, several of these models employ exaggerated variables, which are practically difficult to record. Upcoming versions should be limited to practically relevant regions and validated using scientific results.

#### Enhanced fuel cell performance with biofilm engineering

Considering the significance of EPS in microbial EET as well as its involvement in bacterial growth over electrodes, designing biofilms for improved attachment and EET is only the beginning for MFC. In MFCs, *S. oneidensis* MR-1, a transcriptional shuttle may lower Mn (IV) and Fe (III) ions and generate electricity. Techniques used in *S. oneidensis* MR-1 to carry out this activity are still unknown. However, multiple MR-1 excision variants of *S. oneidensis* were created and evaluated regarding power generation and oxide-based degradation. Results showed that some important cytochromes were involved in EET activities, to a variable extent, revealing a complicated view of electrochemical reactions to rigid and aqueous materials by *S. oneidensis* MR-1. OmcA and MtrC (exterior layer -OM), decaheme Ccyts in rapid electrochemical reactions to strong metallic compounds and electrode materials in MFCs are engaged throughout EET in *S. oneidensis* MR-1; nevertheless, some other species of the group *Shewanella*, *Shewanella loihica* PV-4, revealed a distinct method for power production (Newton et al., 2009). However, in *S. oneidensis* MR-1, genetically engineered strategies were used to co-express the flavin metabolic enzymes gene pattern ribD-ribC-ribBA-ribE and the metal-reducing culvert biosynthetic pathway genotype array mtrC-mtrA-mtrB, leading to an enhanced EET potential in MFC including a boost in the rated current frequency of approximately 110 percent.

To improve flavin-mediated charge transport, an artificial riboflavin cascade from *Bacillus subtilis* was introduced in *E. coli* to generate excess flavins, and a repellent *S. oneidensis* strain CP2-1-S1 was used as the exo-electrogen to enhance its adherence to the anode material. In this respect, the highly hydrophobic contacts among *S. oneidensis* and the anode, including the abundance of flavins generated by the transgenic *E. coli*, gave *S. oneidensis* an edge above *E. coli* with respect to anode adhesion. The catalyzed current was greater in this deliberately designed anodic multilayer with the changed microbiological population composition. The energy capacity of the xylose-fed MFC seeded with such a designed microbiota showed 6.8 times more than the non - transgenic cultured cells injected (Chignell et al., 2018). Shown in Table 4 are investigations dealing with the aspect of biofilm in microbial fuel cells (MFCs) over the past few years.

**Table 4:** Investigations on the aspect of biofilm in microbial fuel cells (MFCs) over the past few years.

S. No.	Research	Features	References
1	The microalgae multilayer microbial fuel cell was created by combining algal biomass (AB) and a microbial fuel cell (MFC) to improve the device’s functioning enabling nutrients clearance with biofuel synthesis	The ABMFC system removes contaminants faster than the AB or MFC systems separately. Clearance effectiveness of N, P, and COD might approach 95.5, 96.4, and 81.9%, respectively, with the maximum voltage densities of 62.93 mW.m^−2^ and lipids production of 6.26 mg.L^-1.^d^−1^	Yang et al., 2018
2	The viability of increasing EET and associated bioremediation capability by gene editing of *Shewanella oneidensis* MR-1 was investigated	In MFC and potentiostat-controlled electrolytic systems, the modified microbe outperformed the reference microbe in parameters of EET ability, yielding a rated load intensity gain of around 110%	Huang et al., 2019
3	A freshly generated *Geobacter sulfurreducens* 11,501 variant demonstrated that such a variation impairs an amplicon involved in the production of polysaccharides, which attach c-type cytochromes associated with cellular electron transport. Biofilms connected to electrodes were pigmented utilizing a Live/Dead *Bac*Light microbial survivability reagent and photographed	In microbial fuel cells, surface sugars regulate cellular adherence to graphite anode material and energy production	Rollefson et al., 2011
4	Evaluation of the current-generating capabilities of *Shewanella loihica* PV-4 in MFCs and that of well-characterized *S. oneidensis* MR-1. Examination of the involvement of c-cytochromes in external energy transmission	Charge transfer performance in the PV-4 microbial fuel cell reached 26%, but just 16% inside the MR-1 microbial fuel cells. MtrC homologue is the major route of charged particles approaching the anode throughout the current-generating processes of *S. loihica* PV-4 amid anode connected microbial community	Newton et al., 2009
5	*G. sulfurreducens* biofilms developed in water passing devices containing graphite photoanode as charged particle consumer, and a fumarate-like electron acceptor, on another graphite surface. During the power-harvesting phase, removal of pilA or omcZ significantly reduced the energy generation and colony development	Microbial cells developed with fumarate, with no substantial present output have physiological variations between various power-generating biofilms. OmcZ plays an important role in charge transfer from specialized *G. sulfurreducens* microbes to the working electrode	Ueki et al., 2018
6	Laboratory model microbial fuel cell (MFC) infected using irrigated crop soil sample and supplied polymers as carbon and power supply. Microbes that generate power are concentrated in biofilm communities on the anode. Microscopy as well as spectroscopy examination of the microbiota	Using plant material as the power supplier, a microbiota (mostly *Rhizobiales*) supplemented irrigated crop soils power production of approximately 0.3 mA	Ishii et al., 2008
7	The relationship among crucial cytochromes and power generation in *S. oneidensis* MR-1 normal type and genetically altered MFC electrodes was investigated by SEM using the wild-type strain and mutant deficient in c-type cytochromes and protein secretion systems	The very first collection of power intensities acquired using microbial fuel cells connected to bacterial physiology. Attributed to the prevalence of much more microorganisms over the top, a potential framework that increases multilayer development gives greater power generation plus oxide-based elimination efficiencies	Bretschger et al., 2007

Similarly, utilization of incomplete degradation of graphite composite substrate through UV/O_3_ exposure led to surface properties changes, thus enabling improved bacterial growth, greater electron transport velocity, and production of larger voltage intensity *via* MFC. Research findings indicated that *S. oneidensis* MR-1 microbial communities establishment was advanced on UV/O_3_-medicated graphite felt anode and cathode at an electric voltage of 0.3 V vs. Ag/AgCl, with the graphite electrodes subjected to 45 min of UV/O_3_ intervention offering better electrochemical findings and microbial cells connection (Cornejo et al., 2015). In addition, the effect of specific working circumstances affecting biofilm production and nitrogen fixation in three moving-bed biofilm reactors (MBBRs) was studied. To investigate the significance of such attachments, scientists employed genetics and gene suppression techniques, which revealed a complete image of the EET route from bacterium to the anode. However, no reports of genetically modified or biochemical pathways of phytoplankton, as well as their potential productivity in energy cultivation, have been published to date. Nonetheless, increasing the individual’s EPS synthesis using substrate modification techniques has been shown to enhance power generation in phytoplankton (Angelaalincy et al., 2018). Besides its involvement in the complex formation of amylopectin and the formation of new capsules, starch synthase, a glycosyltransferases 5 (GT5) enzyme, has been investigated for its involvement in promoting carbohydrate formation. As of now, there has been no evidence of up-regulation of these polysaccharide-producing enzymes in algae. Thus, gene editing and transcription factors involved in exo-polysaccharide yield in microalgal species will require extensive investigations and are expected to become a successful technique for increased production of electricity in photosynthesizing algal microbial fuel cells (PAMFCs), are more durable than microorganisms fuel cells. As a result, biofilm construction is a vast study field.

## Scaling up of the current density generated by biofilms

Power generation does not increase exponentially with effective contact coverage of the MFC electrodes to meet the needs of elevated activities, which is one of the limitations of BESs. To assist scalability of MFCs, approaches such as lowering oxidation and reducing overpotentials, improving fluid conductance, reducing mass transfer resistances, cutting electrodes distance, using novel gas electrode materials, and layering have been employed (Ieropoulos et al., 2008). Whereas such advancements in MFC architecture and scaling have increased maximal energy output, they have not; however, tackled the current development of electrodes employed in such devices’ fundamental scaling (Dewan et al., 2008; Cheng et al., 2009). For example, scaling up the production of optimum energy in sewage MFC was shown to be quite tightly related to the discharge contact area (a 62% higher in energy by twice the discharge contact area) than the anode contact area (12% higher in current by twice the anode area; Babauta et al., 2012). Similarly, the difference in power increase percentages demonstrates the lack of fundamental information about scaling up MFCs. There is a space for enhancement that might include the use of models built particularly to optimize MFCs (Kato Marcus et al., 2007; Picioreanu et al., 2007). The scaling up of anodes in MFCs and BESs is linked with the EET methods used by EABs. The importance of addressing the unique involvement of every electrode in total energy transmission cannot be overstated. Eventually, these gadgets’ inefficiency in power generation will persist (Babauta et al., 2012).

## Future research directions for electrochemically active biofilms

EAB research is still in its infancy, but there have already been some significant breakthroughs in the field of electron transport processes. A first step may be to investigate a variety of measuring methods for quantifying electron fluxes and archiving in EABs. Electrochemical assays should, ideally, be used in conjunction using visual observations taken in the field to calculate the surface area and density of biofilms as a time-dependent variable (Ter Heijne et al., 2021). Under varying circumstances, this would make it possible to the analysis of bioactive substances per unit of biomass. As an example, Raman spectroscopy (Virdis et al., 2012) and CSLM (confocal laser scanning microscopy; Baudler et al., 2015) might be utilized to find which parts of the cell are important for electron transit and storage. Along this line, UV–visible light spectroscopy can be used to determine the concentration of cytochrome heme (Zhang et al., 2018), and to determine the degree of cytochrome pool decreases (Huang et al., 2019), or fluorescence spectroscopy (Esteve-Núñez et al., 2008). As a result, the redox status of cytochromes may be utilized as a model to forecast current output (Ferreira and Salgueiro, 2018). Similarly, cyclic voltammetry can be employed for determining the intermediate voltage of functional charge carrier cellular constituents (Fricke et al., 2008). Moreover, by utilizing a blend of such approaches, the periodic characteristics of several memory activities can be determined in more details (Ter Heijne et al., 2008).

In MFCs, air access toward the electrolyte and electrode microflora is a new field of study with several possibilities. In this respect, oxygen can break down complex organics into by-products that can generate electricity. It is also important to identify the viability of mutualistic interactions among stringent anaerobes and aero tolerant anaerobes and determine the function of biofilm in protecting the electrode from contamination. A few examples would be studying the EET processes in partly aerobic circumstances. These topics should be the focus of fascinating study in the coming years. Another set of questions arises from employing MEC/BES technology to produce energy and goods utilizing sewerage like a commodity while simultaneously attempting to recover and recycle water. The capacity of EABFs to handle a variety of biological molecules, and the amount of electron donor mineralization, are two key factors in this context. Furthermore, conversion of particulate and complicated natural substances is a key problem that may necessitate pre-processing to achieve significant restoration of electrons and transformation. As systems reach commercially relevant performance, activity in this field will continue to grow. However, numerous uncertainties remain concerning the processes that allow EABFs to transport electrons toward the anode, although much is known about them. The importance of biofilm architecture and its constituents including EPS and nanotubes in regulating electron conductance in EABFs is currently unknown; however, it is crucial to understand how they work and how they may be improved. The use of tiny MFCs and microplate-based fluid chambers in conjunction with voltage and current-sensitive pigments and bio-molecules seems to have the ability to simplify experiments, thus providing a better understanding of the electroactivity of EABF components.

Understanding the architecture of the EABF in association with effective substrate consumption and electron transport might give an insight into the mechanics and development of biofilms. In large-scale systems, factors such as reagent supply and dispersal, as well as fluid velocity may be affected by biofilm characteristics, making bioprocess development and design critical. Therefore, further research is required to greater understanding of these connections between research and practice, and to demonstrate how innovative and modeling techniques may be combined to expand such approaches. Furthermore, it is necessary to develop biocathode systems that are optimal for generating energy and/or obtaining value-added goods. In biocathodes, fundamental issues about electron transport and energy sources remain unanswered. The study of the mechanism of microorganisms activating processes associated with the electrodes by adjusting CO2 by the groups of mixotrophic or chemolithotrophic bacteria is an important field of study at resecting times (Borole et al., 2011).

## Conclusion

In summary, we have demonstrated through this review that MFCs are effective systems to meet increasing energy requirements. Waste materials are utilized to produce fuel in MFCs, thereby lowering pollutant concentration. Numerous sectors have adopted this approach for a variety of applications, including sewage treatment, biodiesel, or biofuel production, as well as biosensors. The efficiency of MFCs must be increased while simultaneously enabling huge quantities of energy generation. In this respect, biofilm engineering might help improve mass transport and electron transfer processes at electrode-electrolyte interfaces. Charge transfer impedance at the electrode-electrolyte interface can be reduced by improving microbial activities and higher electrical conductivity. In addition, customizing biofilms and increasing rates of bio-electrocatalysis could be substantially aided by improved electrode materials and electrode functionalization methods. Furthermore, for bio-electrochemical purposes, genetically modified and synthetic biology techniques to boost the overexpression of genes/proteins engaged in the creation of biofilms, electron transport, and electrocatalysis are intriguing.

## Author contributions

AR, AS, BT, SPan, DL, MN, SPat, TS, RR, and PW: conceived and designed the work. AR, AS, BT, SPan, DL, MN, SPat, TS, RR, HAE, and PW: writing—original draft preparation. AR, AS, BT, SPan, DL, MN, SPat, TS, RR, PW, and MM: formatting, editing according to journal guidelines, and writing—review and editing. All authors contributed to the article and approved the submitted version.

## Conflict of interest

SPat is employed by NatNov Bioscience Private Ltd.

The remaining authors declare that the research was conducted in the absence of any commercial or financial relationships that could be construed as a potential conflict of interest.

The handling editor declared a past collaboration with one of the authors RR.

## Publisher’s note

All claims expressed in this article are solely those of the authors and do not necessarily represent those of their affiliated organizations, or those of the publisher, the editors and the reviewers. Any product that may be evaluated in this article, or claim that may be made by its manufacturer, is not guaranteed or endorsed by the publisher.
